# A Comparison of the Clinical Presentation of Ovarian Hyperstimulation Syndrome in a Partial Molar Pregnancy Case Versus a Fertility Treatment Case

**DOI:** 10.7759/cureus.4718

**Published:** 2019-05-22

**Authors:** Elyssa Cohen, Jennifer L Lanzer, Pardeep Mittal

**Affiliations:** 1 Miscellaneous, Medical College of Georgia at Augusta University Medical Center, Augusta, USA; 2 Obstetrics and Gynecology: Female Pelvic Medicine and Reconstructive Surgery, Medical College of Georgia at Augusta University Medical Center, Augusta, USA; 3 Radiology and Imaging, Medical College of Georgia at Augusta University Medical Center, Augusta, USA

**Keywords:** ovarian hyperstimulation syndrome, partial molar pregnancy, fertility treatment, dilation and evacuation, thyrotoxicosis

## Abstract

Ovarian hyperstimulation syndrome (OHSS) is ovarian enlargement secondary to hormones overstimulating ovarian growth. It can be associated with a spectrum of other clinical findings, including ascites, hemoconcentration, hypercoagulability, and electrolyte imbalances. OHSS most commonly occurs as a complication of treatment with in vitro fertilization medications, such as human chorionic gonadotropin (hCG) or gonadotropin-releasing hormone agonists. OHSS has infrequently been reported to be caused by high hCG levels in complete, partial, or invasive molar pregnancies. The classic signs and symptoms of OHSS include nausea, vomiting, bloating, abdominal pain, tachycardia, tachypnea, and dyspnea. Further positive diagnostic studies for OHSS include enlarged ovaries, ascites, hemoconcentration, hyponatremia, hyperkalemia, and oliguria. OHSS due to molar pregnancies is extremely rare. Suziki et al. performed a literature review in 2014 and describe the eight ever-reported molar pregnancy-associated OHSS cases, three of which were partial molar pregnancies. We present a two-case comparison that first examines an exceptionally rare OHSS case presentation of a 19-year-old female with a partial molar pregnancy that was also complicated by hCG-induced thyrotoxicosis. Following this, we discuss a case of the more classic presentation of OHSS caused by fertility treatments. This case report is of novel interest because we present a case comparison that emphasizes a rare, paradoxical association between OHSS and dilation-evacuation procedures that is important for physicians to be aware of - OHSS is not an adverse event of molar pregnancies that can be eliminated by declining hCG levels after a dilation and evacuation procedure; rather, in a molar pregnancy, OHSS occurs after the dilation and evacuation.

## Introduction

Ovarian enlargement in the setting of ovarian hyperstimulation syndrome (OHSS) most commonly occurs iatrogenically as an adverse effect of in vitro fertilization (IVF) medications, such as human chorionic gonadotropin (hCG) or gonadotropin-releasing hormone (GnRH) agonists. Clinically evident OHSS has an incidence of 1%-5% in patients who undergo assisted reproductive technology, with 0.5%-1.0% of women requiring hospital admission for OHSS management [1]. OHSS has infrequently been reported to be caused by the following etiologies: high human chorionic gonadotropin (hCG) levels in a multifetal pregnancy, high hCG levels in a complete/partial/invasive molar pregnancy, or hypothyroidism with high thyroid stimulating hormone (TSH) levels acting similarly as hCG [2]. The classic signs and symptoms of OHSS include nausea, vomiting, bloating, abdominal pain, tachycardia, tachypnea, and dyspnea. Further positive diagnostic studies for OHSS include enlarged ovaries, ascites, hemoconcentration, hyponatremia, hyperkalemia, and oliguria [3].

The pathogenesis of OHSS involves an hCG-mediated vascular endothelial growth factor (VEGF) response that causes increased vascular permeability and arteriolar vasodilation [3]. This allows for a fluid shift from the intravascular to extravascular compartments, creating a state of hypovolemic hyponatremia with hemoconcentration [3].

Women affected by OHSS also have an increased incidence of thromboembolism as compared to non-in vitro fertilization pregnant women - 1.68% versus 0.02%, respectively [3]. OHSS can be life-threatening, but in most cases, it is self-limiting when hCG levels decrease and ovary stimulation ceases [3]. OHSS due to molar pregnancies is extremely rare. Suziki et al. performed a literature review in 2014 and describes the eight ever-reported molar pregnancy-associated OHSS cases, three of which were partial molar pregnancies {2}. In this case report, we present a two-case comparison that first examines a much rarer OHSS case presentation of a partial molar pregnancy that was also complicated by hCG-induced thyrotoxicosis, and then we discuss the more classic presentation of OHSS caused by fertility treatments.

## Case presentation

Case 1: OHSS due to a partial molar pregnancy

A 19-year-old gravida 2 para 1 female, approximately 10 weeks pregnant dated by her last menstrual period, with a history of one prior spontaneous abortion, presented to the emergency department with sharp lower abdominal pain, nausea, emesis, and a racing heart. She had been experiencing the abdominal pain for a week and it had progressively worsened. She denied vaginal bleeding, leakage of fluid, and discharge. She did not know her last menstrual period and had not yet received an ultrasound for this pregnancy.

**Figure 1:**
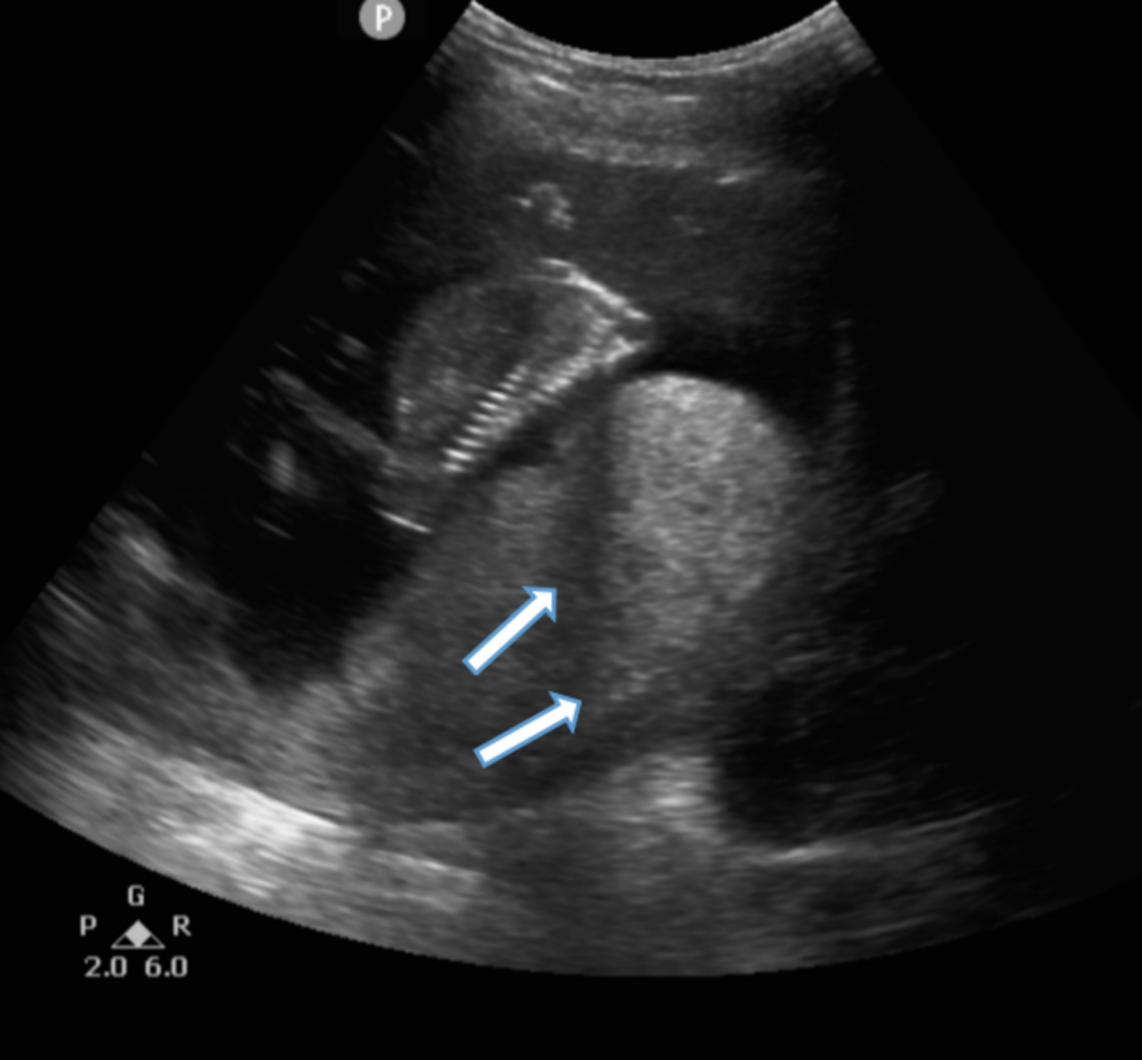
Bedside ultrasound showing intrauterine pregnancy. Bedside ultrasound of a 19-year-old gravida 2 para 1 female showing an intrauterine pregnancy with cystic tissue (arrows), posterior placenta, and present fetal heart tones (waveforms not present in the figure above).

On physical exam, she was tachycardic (144 beats per minute), normotensive, had no proptosis or lid lag, thyroid was non-tender and non-enlarged, and normal S1 and S2 with no murmurs. Bedside ultrasound showed an intrauterine pregnancy with cystic tissue, posterior placenta (Figure 1), and present fetal heart tones. Laboratory results showed a high beta hCG (greater than 1.3 million mIU/mL), low TSH (0.007 mcIU/mL), and high free thyroxine (T4) (2.43 ng/dL). It was concluded that she had a partial molar pregnancy with hCG-induced thyrotoxicosis.

**Figure 2:**
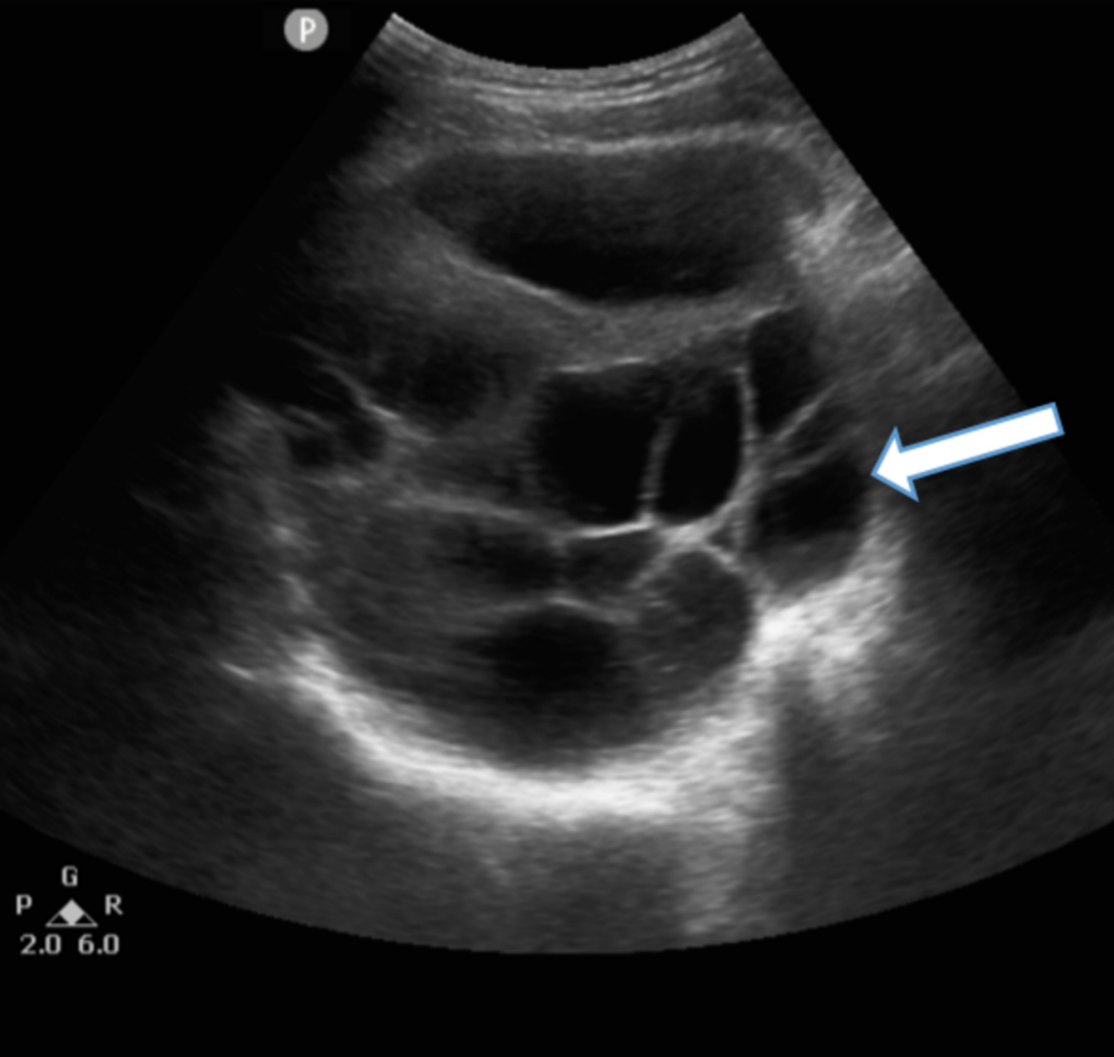
Transvaginal ultrasound displaying a cystic ovary. One of the bilaterally enlarged cystic ovaries (arrow) is shown on this transvaginal ultrasound of a 19-year-old gravida 2 para 1 female.

A transabdominal ultrasound calculated a gestational age of 15.2 weeks based on biparietal diameter, head circumference, abdominal circumference, and femur length. Fetal abnormalities were noted, including tachycardia (221 beats per minute) and hydrocephalus. Bilaterally enlarged cystic ovaries were identified by transvaginal ultrasound (Figure 2); the right ovary measured 13.5 x 11.1 x 8.8 cm (volume of 690.5 mL) while the left ovary measured 13.5 x 13 x 10.8 cm (volume of 992.4 mL) and theca lutein ovarian cysts were seen on both ovaries. Fluid was identified in the right fallopian tube and it measured 10 x 7 cm.

The patient received propranolol and propylthiouracil for medical management of her thyrotoxicosis. When she was stable for surgery, she underwent a routine dilation and evacuation. The pathology evaluation of the placental tissue revealed abnormal chorionic villi with scalloping and occasional fibrotic villi. Fetal parts were noted grossly. The findings in combination with the patient’s clinical presentation confirmed the diagnosis of a partial molar pregnancy. Her beta hCG trended down appropriately during her hospital stay, to a low of 110,097 mIU/mL at discharge. At discharge, laboratory results demonstrated a decreased free T4 to a normal level of 1.32 ng/dL and normal total triiodothyronine of 1.13 ng/mL. She remained normotensive with a normal rate and rhythm. She was counseled on the need to follow beta hCG levels weekly until the value was persistently zero, as well as her increased risk of gestational trophoblastic neoplasia. She received methotrexate to treat any remaining molar tissue and was discharged with a one-week follow-up.

**Figure 3:**
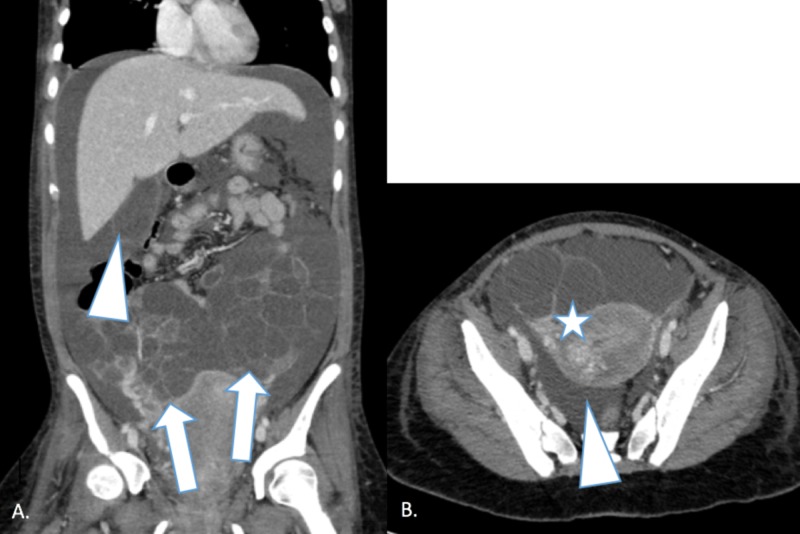
Computed tomography of the thorax, abdomen, and pelvis in a 19-year-old female showing findings of severe (grade 4) ovarian hyperstimulation syndrome. Image A - coronal computed tomography image showing multiple large bilateral cystic structures in the pelvis (arrows) with intra-abdominal and pelvic free fluid (arrowhead). Image B – axial computed tomography image showing heterogeneous enhancing material in the uterus (contained products of pregnancy) (inferior to star). These findings in images A and B are consistent with a nonviable partial molar intrauterine pregnancy and severe (grade 4) ovarian hyperstimulation syndrome.

She returned to the emergency department two days after discharge with vaginal bleeding, abdominal pain, abdominal distension, right lower back pain, and shortness of breath, with a minimally productive cough. Her thyroid studies were within normal limits. Her white blood cell (WBC) count was 21 thousand/mm^3^, platelets were 803 thousand/mm^3^, and beta hCG was 64,950 mIU/mL (which had trended down from the value at the time of her previous hospital discharge). On physical exam, she was afebrile, normotensive, and tachycardic (110 beats per minute). She did not have hemoconcentration, abnormal coagulation studies, electrolyte abnormalities, or oliguria. A chest X-ray demonstrated a moderate right pleural effusion and computed tomography (CT) of the thorax, abdomen, and pelvis showed large multilocular cystic ovaries with the right ovary measuring 7.9 x 11.4 x 13.5 cm, the left ovary measuring 13.8 x 9.0 x 13.0 cm, and a significant amount of intra-abdominal pelvic fluid (Figure 3). These findings were consistent with severe (grade 4) ovarian hyperstimulation syndrome. A right thoracentesis removed 600 cc of pleural fluid while paracentesis collected 2.5 L of fluid, providing her with symptomatic improvement. She received intravenous fluids, pain control, and 25% intravenous albumin followed by diuresis with furosemide. Four days after the first paracentesis, she needed a second paracentesis that evacuated 2.2 L of ascitic fluid. Later that day, she was determined stable for discharge, and her beta hCG was 12,791 mIU/mL. Unfortunately, the patient has been lost to follow-up after discharge.

Case 2: OHSS due to fertility treatment

A 30-year-old gravida 1 para 1 female with a two-year history of unexplained infertility was undergoing IVF treatment. She had a baseline ultrasound performed 12 days before fertility drugs were initiated, and this ultrasound demonstrated a normal uterus and normal-sized ovaries. The right ovary measured 2.7 x 1 cm and the left 2.3 x 1.8 cm. She began ovarian stimulation with letrozole injections. She then underwent a standard ultrasound-guided transvaginal oocyte retrieval. The evening after the oocyte retrieval, she reported to the emergency department with severe abdominal pain (worst in the right lower quadrant), two episodes of near syncope during the day, lightheadedness, dizziness, minimal vaginal spotting, and a self-reported fever. On physical exam, she was afebrile, tachycardic (111 beats per minute), normotensive (107/75 mmHg), but in no acute distress. A genitourinary exam was deferred at this time.

Transvaginal and abdominal ultrasounds were obtained to evaluate for ovarian hyperstimulation syndrome and ovarian torsion. Ultrasound results demonstrated an enlarged right ovary, measuring 5.0 x 4.6 x 5.3 transvaginally (volume of 87.25 mL) and an enlarged left ovary (volume of 47.8 mL). A spectral Doppler analysis demonstrated normal arterial and venous blood flow to both ovaries. Small volume intrapelvic fluid was also visualized. It was determined this patient had mild grade 2 ovarian hyperstimulation syndrome with no evidence of ovarian torsion or acute GI pathology.

**Figure 4:**
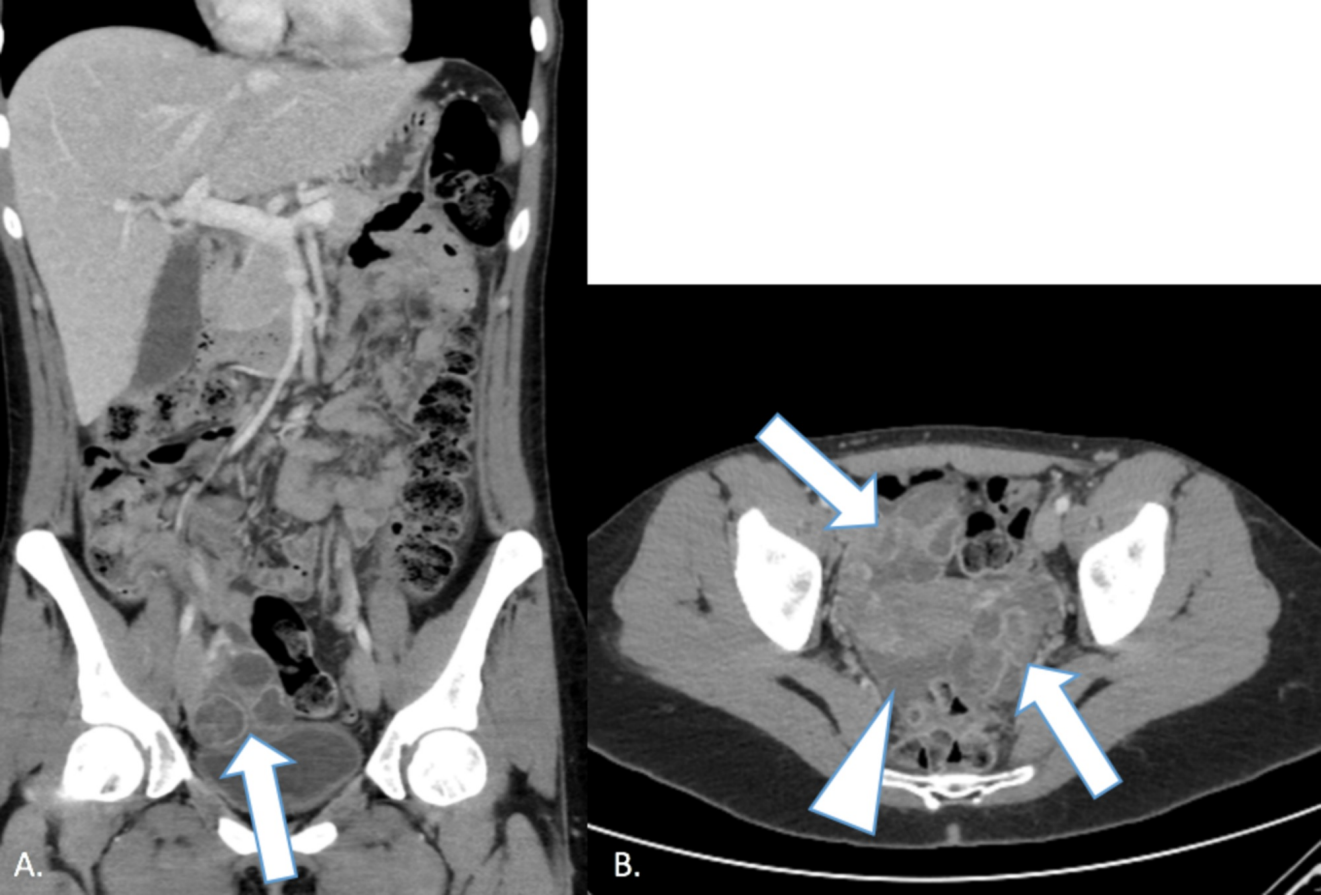
Computed tomography imaging of a 30-year-old female with a recent history of in vitro fertilization treatment showing findings consistent with mild grade 2 ovarian hyperstimulation syndrome. Image A is a coronal computed tomography image and image B is an axial computed tomography image that shows the patient's uterus with bilaterally enlarged cystic ovaries (arrows) and a small amount of fluid in the pelvis (arrowhead). These findings are consistent with the diagnosis of mild grade 2 ovarian hyperstimulation syndrome, as diagnosed on prior ultrasound imaging, with no other concurrent abdominal pathologies.

She did not have hemoconcentration, abnormal coagulation studies, electrolyte abnormalities, or oliguria. She received albumin 25% with 200 cc bolus followed by albumin 5%, intravenous fluids, and intravenous pain control. She had a leukocytosis of 15.3 thousand/mm^3^, which is considered acceptable after oocyte retrieval. She continued to have persistent nausea and abdominal pain, and, therefore, a CT of the abdomen and pelvis with contrast was obtained to evaluate other possible etiologies. The CT scan showed a small volume of free pelvic fluid and bilateral enlarged ovaries with multiple follicles (Figure 4) but no evidence of acute gastrointestinal pathology.

The patient was discharged in stable condition with good pain control and instructions to follow up with her obstetrician-gynecologist in one week.

## Discussion

Ovarian hyperstimulation syndrome is classically defined by a spectrum of findings, including ovarian enlargement, ascites, hemoconcentration, hypercoagulability, and electrolyte imbalances. Severe OHSS has serious complications such as pleural effusion, acute renal failure, and venous thromboembolism [4]. OHSS is classified into mild grade 1/2, moderate grade 3, and severe grade 4/5/6. The classification system is based on symptoms, imaging, and test results. Below are the different classifications [5]:

1) Mild OHSS

 - Grade 1: Abdominal distention and discomfort

 - Grade 2: Grade 1 plus nausea, vomiting, ovarian size of 5-12 cm, with or without diarrhea

2) Moderate OHSS

 - Grade 3: Mild OHSS plus imaging evidence of ascites

3) Severe OHSS

 - Grade 4: Moderate OHSS plus clinical evidence of ascites, with or without hydrothorax

 - Grade 5: Additional hypovolemia, hemoconcentration (hematocrit > 45%), coagulation abnormalities, and oliguria

 - Grade 6: Hemoconcentration (hematocrit > 55%), anuria, renal failure, venous thrombosis, and adult respiratory distress syndrome

Mild OHSS occurs in 20%-30% of patients who undergo controlled ovarian stimulation, but it rarely becomes clinically evident and does not require management [3,6]. Moderate OHSS is reported in 2%-4% of patients undergoing ovulation induction and severe OHSS in 0.1%-0.5% [5]. In the cases we presented, the patient with a partial molar pregnancy developed severe OHSS grade 4 and the patient receiving fertility treatments developed mild OHSS grade 2.

OHSS due to ovulation induction is classified as early-onset OHSS or late-onset OHSS. Early-onset OHSS is caused by exogenous hCG used to stimulate ovarian follicular growth. Late-onset OHSS occurs once an embryo is implanted and the woman makes endogenous hCG from the ongoing pregnancy [7]. The OHSS case we present that resulted from controlled ovarian stimulation is an example of early-onset OHSS.

Molar pregnancies occur one in every 2,000 pregnancies [8]. They result from abnormal fertilization and abnormal trophoblastic proliferation [8]. Molar pregnancies are characterized by elevated beta hCG levels and theca lutein cysts causing large ovaries [6]. Two types of molar pregnancies exist, a complete mole (46 XX or XY) and a partial mole (69 XXY, XXX, or XYY) [9]. A partial mole is also defined by containing fetal parts, whereas a complete mole does not contain fetal parts [9].

We present an exceptionally rare case of a 19-year-old female with a partial molar pregnancy, thyrotoxicosis, and OHSS. Suziki et al. completed a 2014 literature review describing the eight ever-reported molar pregnancy OHSS cases, three of which were partial molar pregnancies [2]. Of these three partial molar OHSS cases, only one was stated to have occurred with concurrent thyrotoxicosis [2]. Of these molar pregnancies, OHSS occurred during weeks seven to 16 of gestation, with a median gestational week of 12 [2]. In our case, the patient manifested with OHSS at approximately gestational Week 11 (she presented to the ED with her partial molar pregnancy at a patient-reported approximate gestational age of 10 weeks and then returned to the ED eight days after that initial presentation and was diagnosed with OHSS).

Suziki et al. discuss how seven of the eight molar pregnancy OHSS cases underwent a dilation and evacuation procedure (D&E), and all seven of these patients had the most severe signs and symptoms of OHSS after the D&E [2]. The median peak of OHSS symptoms was 7.5 days after the D&E, with a range of three to 14 days. In our case, our patient returned to the hospital six days after her D&E with severe abdominal pain, abdominal distension, right lower back pain, and shortness of breath. She had a pleural effusion and ascites requiring a thoracentesis and paracentesis respectively. This presentation highlights that OHSS associated with molar pregnancies most often does not occur at the patient’s peak hCG level [2]. Rather, these patients typically present with OHSS when their hCG is already trending down after their D&E procedure [2]. The pathophysiology behind this paradoxical association is not known. It suggests an important association of OHSS with VEGF, an hCG-mediated growth factor also involved with pre-eclampsia, another disease of pregnancy that can have a postpartum presentation. There has been one case report of a patient with markedly increased VEGF after the D&E for a partial molar pregnancy, and this increased VEGF level superseded a clinical worsening of her OHSS [5]. The pathophysiology of OHSS may also be related to other factors as well, such as inflammatory cytokines, luteinizing hormone, estradiol, and the renin-angiotensin system [2].

## Conclusions

In conclusion, we have presented two cases of ovarian hyperstimulation syndrome. The first case portrays the extremely rare possibility of a partial molar pregnancy causing OHSS, and the second case exemplifies the most common etiology of OHSS, which is caused by medications used for ovarian stimulation for IVF. It is highly probable that most obstetricians and gynecologists will encounter molar pregnancies during their career, so it is important for physicians to be aware of the signs, symptoms, and complications associated with molar pregnancies. In this report, we highlight one of the rarer complications - OHSS. Of distinctive importance, it is critical for physicians to recognize that the risk of OHSS associated with molar pregnancy is not resolved at the time of dilation and evacuation; rather, in molar pregnancy, OHSS most commonly presents after D&E. Our purpose in presenting these cases is to improve physician awareness of this complication in order to influence patient counseling and clinical management on this risk of OHSS in molar pregnancy.
